# Network science meets respiratory medicine for OSAS phenotyping and severity prediction

**DOI:** 10.7717/peerj.3289

**Published:** 2017-05-09

**Authors:** Stefan Mihaicuta, Mihai Udrescu, Alexandru Topirceanu, Lucretia Udrescu

**Affiliations:** 1 Department of Pulmonology, Victor Babes University of Medicine and Pharmacy, Timisoara, Romania; 2 Department of Computer and Information Technology, University Politehnica of Timisoara, Timisoara, Romania; 3 Faculty of Pharmacy, Victor Babes University of Medicine and Pharmacy, Timisoara, Romania

**Keywords:** Network science, Sleep apnea, Phenotypes, Prediction score, Prediction specificity

## Abstract

Obstructive sleep apnea syndrome (OSAS) is a common clinical condition. The way that OSAS risk factors associate and converge is not a random process. As such, defining OSAS phenotypes fosters personalized patient management and population screening. In this paper, we present a network-based observational, retrospective study on a cohort of 1,371 consecutive OSAS patients and 611 non-OSAS control patients in order to explore the risk factor associations and their correlation with OSAS comorbidities. To this end, we construct the Apnea Patients Network (APN) using patient compatibility relationships according to six objective parameters: age, gender, body mass index (BMI), blood pressure (BP), neck circumference (NC) and the Epworth sleepiness score (ESS). By running targeted network clustering algorithms, we identify eight patient phenotypes and corroborate them with the co-morbidity types. Also, by employing machine learning on the uncovered phenotypes, we derive a classification tree and introduce a computational framework which render the Sleep Apnea Syndrome Score (SAS_Score_); our OSAS score is implemented as an easy-to-use, web-based computer program which requires less than one minute for processing one individual. Our evaluation, performed on a distinct validation database with 231 consecutive patients, reveals that OSAS prediction with SAS_Score_ has a significant specificity improvement (an increase of 234%) for only 8.2% sensitivity decrease in comparison with the state-of-the-art score STOP-BANG. The fact that SAS_Score_ has bigger specificity makes it appropriate for OSAS screening and risk prediction in big, general populations.

## Introduction

Obstructive Sleep Apnea Syndrome (OSAS) is a serious clinical disorder caused by abnormal breathing pauses that occur during sleep; this results in sleep fragmentation and excessive daytime somnolence (Simon & Collop, 2012; Fischer et al., 2012; Lévy et al., 2014). There are studies reporting the epidemic incidence of OSAS, with worrying increasing rates over the last 20 years (Young, Peppard & Gottlieb, 2002; Punjabi, 2008; Peppard et al., 2013). If not properly diagnosed and treated, OSAS increases the morbidity and perioperative risks (Memtsoudis, Besculides & Mazumdar, 2013; McNicholas, Bonsignore & of EU Cost Action B26, 2007; Rossi, Stradling & Kohler, 2013; Utriainen et al., 2013; Sánchez-de-la Torre, Campos-Rodriguez & Barbé, 2013). Indeed, when it remains undetected, OSAS rapidly creates serious cardiovascular, respiratory and nutritional problems (McNicholas, Bonsignore & of EU Cost Action B26, 2007; Yaggi et al., 2005; Bakker, Montesi & Malhotra, 2013). Therefore, it is essential that OSAS be detected at an early stage, which can only be achieved through preventive actions such as extensive population screening (Pelletier-Fleury et al., 2004).

Apnea severity is indicated by the Apnea-Hypopnea Index, *AHI*; this represents the number of breathing pauses of at least 10 s, recorded over one hour of sleep. As such, any patient can be classified in one of the following AHI categories: normal or low-risk apnea (*L*) for *AHI* < 5, mild sleep apnea (*Mi*) for 5 ≤ *AHI* < 15, moderate sleep apnea (*Mo*) for 15 ≤ *AHI* < 30, and severe sleep apnea (*Se*) when *AHI* ≥ 30.

Usually, polysomnography (PSG) is used as the reference method (i.e., gold standard) of OSAS diagnosis, which is based on measuring *AHI*. However, PSG is expensive, time-consuming, and generally not adequate for population screening (Simon & Collop, 2012; Schlosshan & Elliott, 2004). Since OSAS has a significant prevalence and the PSG-based exhaustive investigation is not feasible when screening a large population, OSAS predictors are preferred for monitoring (Udrescu et al., 2014; Topirceanu et al., 2014).

In current medical practice, there are three major predictive models based on questionnaires, namely Berlin, STOP, and STOP-BANG (Netzer et al., 1999; Silva et al., 2011; Farney et al., 2011; Chung et al., 2008a; Chung et al., 2008b). Published studies indicate STOP-BANG as the best available predictive score, due to its high sensitivity: 83.6% for *AHI* > 5, 92.9% for *AHI* > 15, and 100% for *AHI* > 30. However, STOP-BANG has a low specificity (56.4% for *AHI* > 5, 43% for *AHI* > 15, and 37% for *AHI* > 30) (Chung et al., 2008a; Chung et al., 2008b; Chung et al., 2014) which prevents the usage of this score for population screening. Although there are notable attempts for improving STOP-BANG’s specificity (Chung et al., 2014), they are mainly targeting narrow-type cohorts such as perioperative patients.

Consequently, our paper aims at analysing the general case, (i.e., with all patient categories from a given geographical area being taken into account for screening), and not just some specific cohorts. To this end, our research is underpinned by a complex network perspective on uncovering OSAS phenotypes. Indeed, network science is already successfully used in medicine at the disease-level (Barabási, 2011), including respiratory applications (Diez, Agustí & Wheelock, 2014; Faner et al., 2014; Divo et al., 2015). Our network-based approach on OSAS risk factors allows for better, more accurate OSAS phenotype identification, which in turn leads to a new predictive score (*SAS*_Score_). In comparison with the state-of-the-art, our OSAS risk prediction score achieves significantly better specificity in predicting actual AHI categories, which makes our *SAS*_Score_ very appropriate for screening big populations as part of preventive medicine programs.

## Methods

Network Medicine has received a lot of attention during the last decade (Menche et al., 2015; Goh et al., 2007; Barabási, 2007; Vidal, Cusick & Barabasi, 2011); this trend is fuelled by the fact that complex network science can bring significant advances in various medical fields like genomics (Sharma et al., 2013; Rozenblatt-Rosen et al., 2012), drug-target interaction (Yıldırım et al., 2007), or cell metabolism (Barabasi & Oltvai, 2004; Han, 2008). Consequently, it has been recently suggested that network medicine can be also used for addressing important problems in respiratory medicine (Faner et al., 2014; Divo et al., 2015).

### Description of databases

In order to use complex network tools for OSAS research, we need real-world OSAS patient datasets. Unfortunately, OSAS patients datasets are scarce and not public; such a situation is justified by multiple aspects: big data techniques were only recently considered as tools for respiratory medicine and OSAS, all patients must undergo hospital polysomnography (which entails a complex, expensive and time-consuming process), while coordinated research efforts for gathering data were only recently introduced.

For instance, the biggest such OSAS database, namely European Sleep Apnea DAtabase—ESADA (Hedner et al., 2011), is not public and it has gathered data from 15,956 patients in 24 sleep centers from 16 countries since 2007. Also, a recent OSAS study (Marti-Soler et al., 2016) where the validation is similar to our approach uses only one (private) validation database, comprising 1,101 patients (Santos-Silva et al., 2012).

As a result, in order to perform network investigation on OSAS, we built our own Apnea Patients Database (APD) consisting of consecutive patients with suspicion of sleep breathing disorders, which were evaluated at Victor Babes Regional Hospital in Timisoara (Western Romania) between March 2005 and March 2012 under the supervision of the hospital’s Ethics Committee (internal briefing note no. 10/12.10.2013). At the initial visit, the study protocol was clearly explained to obtain the patient’s consent and the acceptance of referral physicians. Subsequently, respiratory polygraphy was performed using both Philips Respironics’s Stardust polygraph (2005) and MAP’s POLY-MESAM IV (1998). PSG was carried out with Philips Respironics’ Alice 5 Diagnostic Sleep System, according to the appropriate guidelines (Rechtschaffen & Kales, 1968). The polygraphy was performed both at home and at the hospital, whereas PSG measurements were performed at the hospital under medical supervision. To preserve the information accuracy, all collected data were carefully verified; throughout this process, we have ensured complete data confidentiality. Our observational, retrospective study employs only standardized non-invasive procedures that exclude all useless investigations. Moreover, visits did not entail additional effort for the patients or supplemental budget for the clinic.

**Figure 1:**
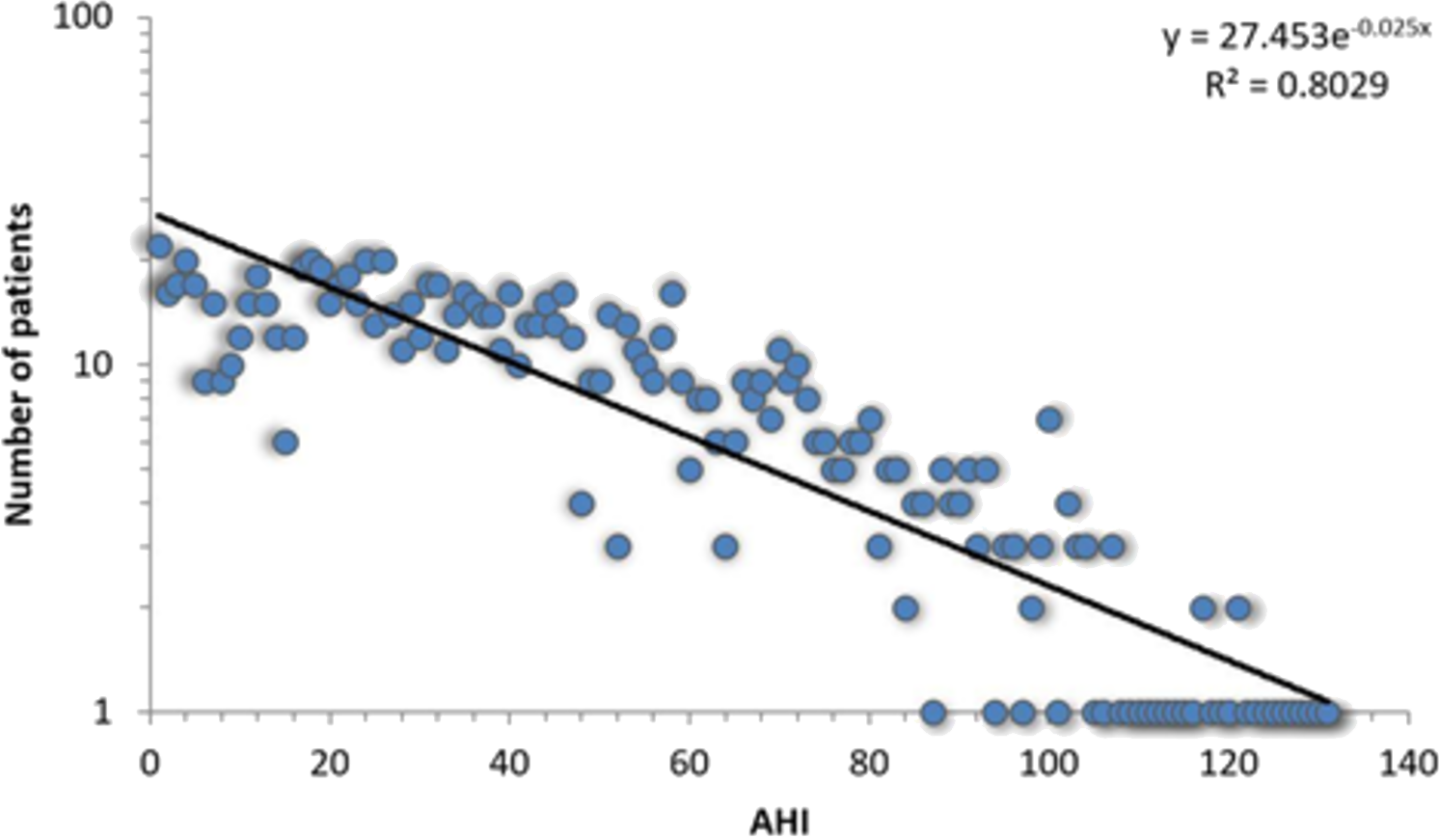
Log-linear histogram representing the number of patients with a certain Apnea-Hypopnea Index (*AHI*) value, along with the corresponding distribution fit. The best fit is the exponential function *f*(*x*) = 27.453⋅*e*^−0.025*x*^.

All 1,371 patients that completed the sleep study protocol and signed informed consent are included in the APD, each with corresponding 108 breathing parameters and anthropometric measurements. The APD distribution of measured *AHI* is presented in Fig. 1.

**Figure 2:**
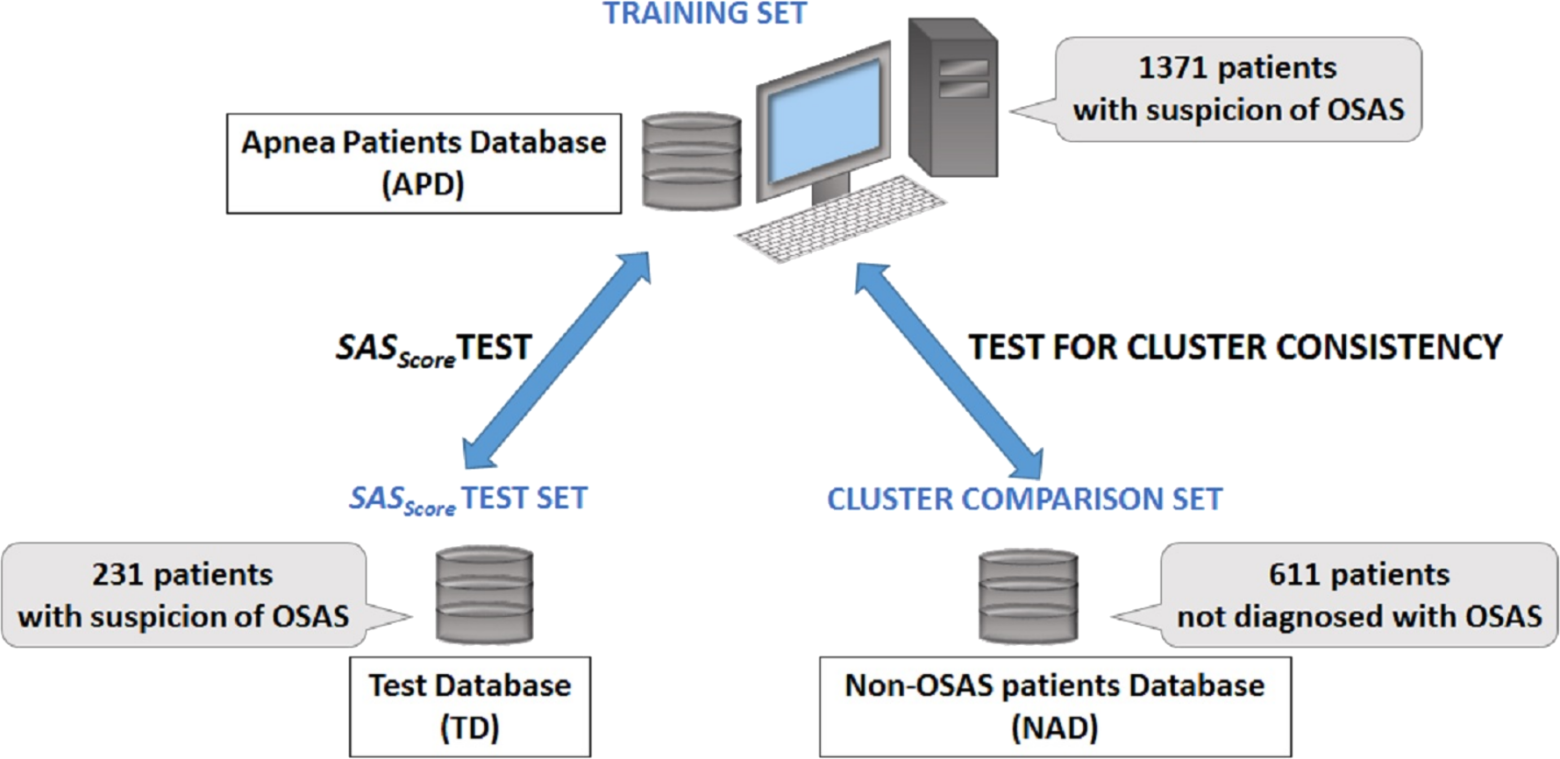
Description of databases used in our research. The main Apnea Patients Database (APD), comprising 1,371 consecutive patients which arrive at the hospital with suspicion of OSAS between 2005 and 2012, is used to build patient phenotypes and to render the *SAS*_Score_. The distinct Test Database (TD), comprising 231 consecutive patients which arrive at the hospital with suspicion of OSAS in 2013, is used to verify the sensitivity and specificity of predicting patient’s *AHI* and OSAS categories. The Non-OSAS patients Database (NAD) uses consecutive assessed people whom are not diagnosed with OSAS during the spring 2015—summer 2016 period, in order to test for cluster consistency (i.e., compare how risk factors converge in clusters for OSAS patients in comparison with people without OSAS).

In order to verify if there is any difference between apnea and non-apnea populations in terms of how risk factors associate and converge, we built a 611 people non-OSAS database NAD (using the same procedure as for the APD). Also, to evaluate the prediction score derived from our study, we gathered a distinct test database TD (over a distinct period of time: the fall of 2013) consisting of 231 patients by following the same procedure. Figure 2 presents the distinct roles of our three databases, as well as the relationship between them.

### Analysis of APD and TD

As patients within TD are used to validate our OSAS prediction with *SAS*_Score_, which was obtained by processing patients from APD, we analyse if the distribution of parameters in TD is not too close to the corresponding distributions in APD. Such an investigation is required considering that, although data for the two databases were gathered over distinct periods of time, all measurements were performed in a given geographical region.

To this end, we present the distributions of the most relevant parameters in our research (Age *A*, Body Mass Index *BMI*, Neck Circumference *NC*, High Blood Pressure *HBP*, and Epworth Sleepiness Score *ESS*) within the validation population (TD) and the apnea patients database (APD) in Table 1 under the form of measured averages and their corresponding standard deviations, as well as Gini coefficients. We rely on Gini coefficients for a quantitative measure of data dispersion.

**Table 1:** AHI and relevant risk factor parameters distribution in the Apnea Patients Database (APD) and Test Database (TD), given as average values plus standard deviation, as well as Gini coefficients. We only considered boolean values for the High Blood Pressure (i.e., if the patient has high blood pressure or not); in this case, we provided the percentages of people with high blood pressure.

Parameter	APD		TD	
	Average	Gini	Average	Gini
*AHI*	40.04 ± 27.83	0.388	44.39 ± 26.13	0.334
*BMI*	33.01 ± 7.94	0.576	32.62 ± 7.41	0.468
*A* (yrs.)	51.73 ± 12.44	0.539	52.01 ± 13.61	0.397
*NC* (cm.)	42.01 ± 9.94	0.51	42.64 ± 5.12	0.482
*HBP*	67.47%	N/A	64.93%	N/A
*ESS*	10.63 ± 5.38	0.362	11.38 ± 5.19	0.422

**Figure 3:**
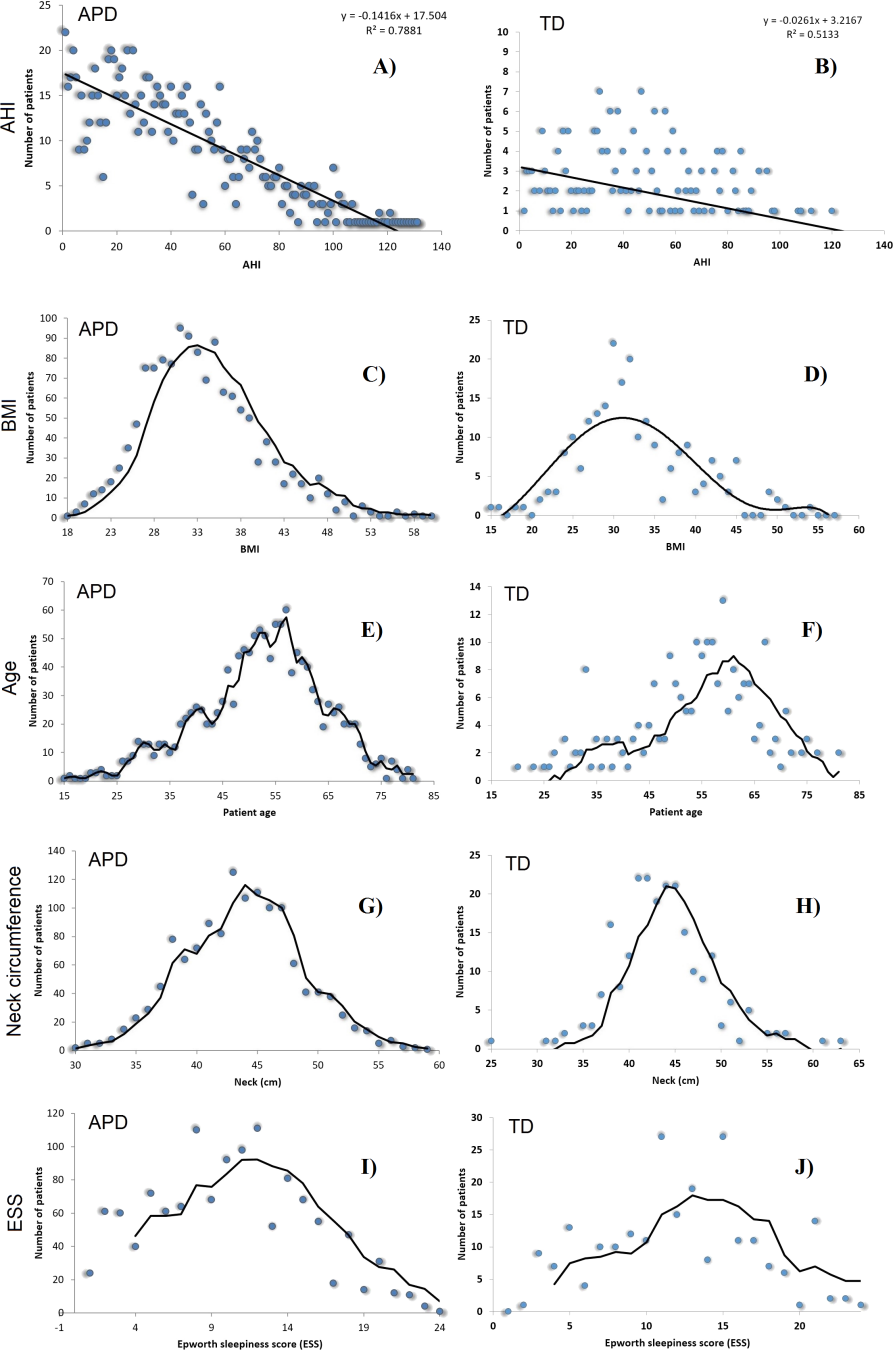
*AHI* distributions in APD (A, C, E, G, I) and TD (B, D, F, H, J), as well as the normal distributions of *BMI*, Age *A*, Neck Circumference *NC* and *ESS*, for patients in both APD and TD.

We also provide a visual comparison of *AHI* and relevant risk factor parameters distributions in APD and TD (see Fig. 3). All these results show that data were randomly gathered, so that the main parameters are normally distributed. However, Gini coefficients (especially for *A*, *BMI* and *ESS*) indicate an important difference between APD and TD distributions. Moreover, Fig. 3 shows a significantly different *AHI* histogram for TD in comparison with APD. As such, in APD there are many patients with *AHI* >120, whereas in TD there is none such patient. Also, in APD the largest number of patients associated to an *AHI* value correspond to *AHI* values <20; in contrast, in TD, the largest number of patients with a given *AHI* value correspond to *AHI* values around 40.

### Building the patient network

An unweighted network is a graph (*V*, *E*), which consists of a set of vertices (or nodes) *V* and a set of edges (or links) *E* that represent connections $ \left[ v,w \right] \in E$ between certain pairs of vertices *v*, *w* ∈ *V*. We build the unweighted Apnea Patients Network (APN), by assigning vertices and edges: each node corresponds to a distinct patient in our OSAS patients database APD, while an edge (link) is created between two vertices if there is a risk factor compatibility between the patients represented by the two vertices (nodes).

The risk factor compatibility is a binary function *f*_*RFC*_ ∈ {0, 1} (0 means incompatibility and 1 means compatibility) based on six parameters with high relevance for OSAS: age, gender, BMI, neck circumference, blood pressure (systolic and diastolic), and Epworth Sleepiness Score. We build our APN by considering that *f*_*RFC*_ = 1 if at least four out of six parameters are identical; otherwise *f*_*RFC*_ = 0.

The six parameters are selected from the pool of all relevant risk factors (all measured parameters can be found in the Supplemental Information 1 because they can be measured easily and objectively; such objective measurements can be performed anywhere, and are widely accepted in the medical literature (Lévy et al., 2014). In contrast, other scores consider snoring and witnessed apnea episodes as factors, but these are parameters which cannot be observed or measured objectively.

**Figure 4:**
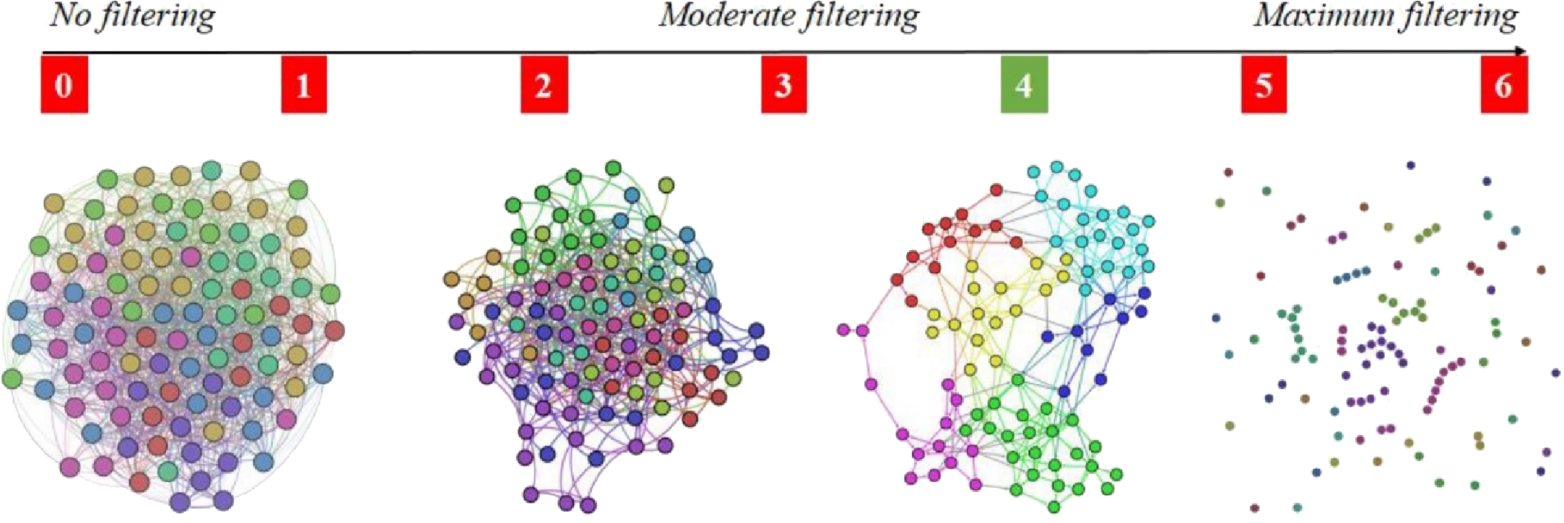
Apnea Patients Network (APN) edge filtering, by considering different definitions for *f*_*RFC*_ = 1, when we adopt the *x*-out-of-6 criteria (*x* = 1, 2, 3, 4, 5, and 6). The visual result indicate *x* = 4 as the best solution, because the edge density is convenient for rendering topological clusters with energy model layouts. Other values for *x* will generate too dense or too sparse networks.

The reason for adopting the 4-out-of-6 criterion is that it assures the right amount of link density in the APN, meaning that there are enough links so that the APN is connected, but not too many links so that communities (i.e., clusters) can be rendered with energy model layouts (Noack, 2009). As Fig. 4 shows that the 4-out-of-6 link filtering represents the best alternative, we use this criterion to build the APN. To the best of our knowledge, this link filtering procedure is original and has not been used before in such network-based approaches.

**Figure 5:**
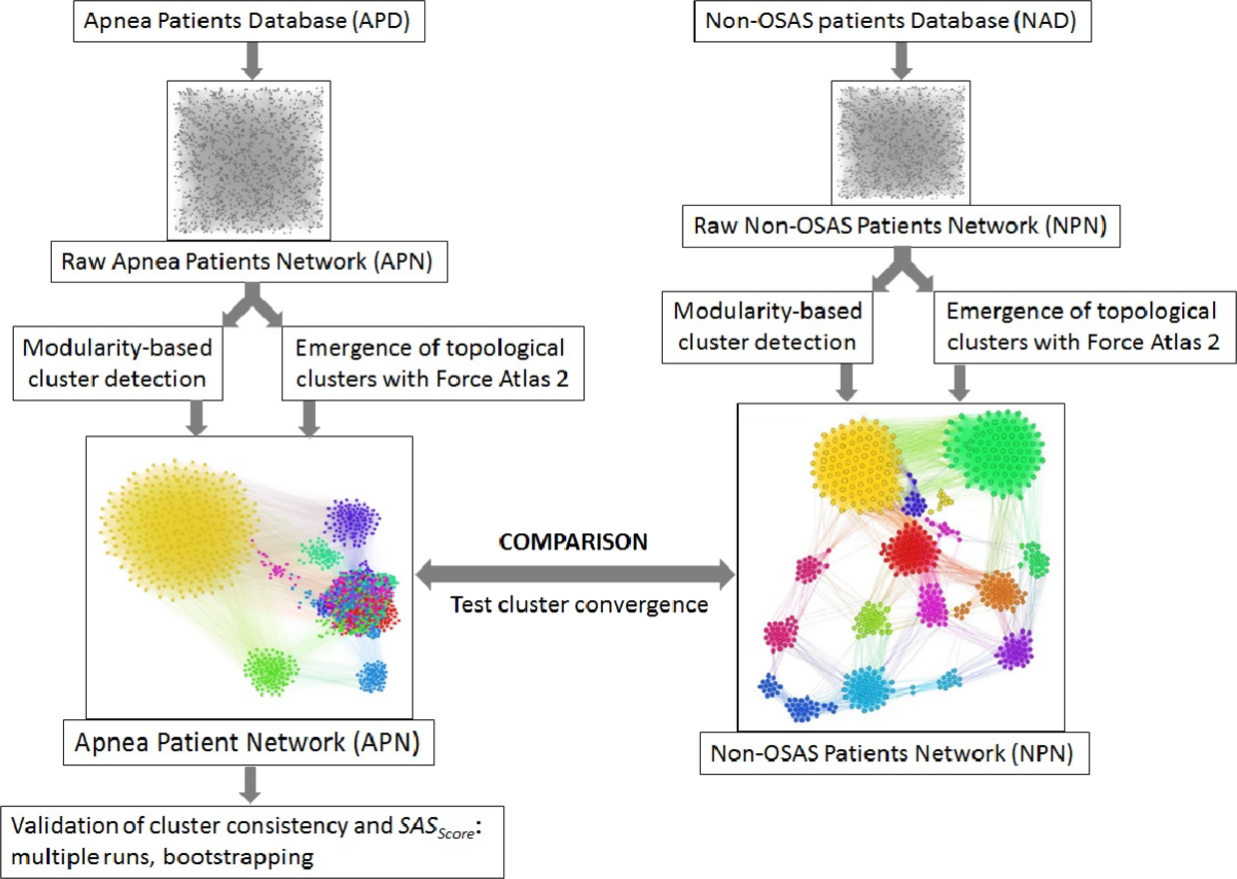
Overview of the proposed dual clustering methodology (modularity classes represented with distinct colors and topological clusters emerged from running the force directed layout Force Atlas 2), along with testing cluster formation against non-OSAS control patients (i.e., showing that risk factors converge differently for OSAS and non-OSAS patients), cluster consistency and *SAS*_Score_ validation.

### APN clustering

We clustered the APN by using a dual clustering methodology: energy-model layouts plus modularity classes, similar to the approach from (Udrescu et al., 2016; Udrescu et al., 2014). Energy-models are force directed network layout algorithms, namely visual tools that assign certain positions in the Euclidian space to both nodes and edges (Noack, 2009). To this end, we used the Force Atlas 2 algorithm (Jacomy et al., 2014) as the network layout; this new layout is very effective in clustering various types of complex networks, as it is based on previous theoretical foundations of force directed attraction–repulsion algorithms (Fruchterman & Reingold, 1991; Noack, 2003). Indeed, Force Atlas 2 is clustering complex networks by producing well-defined topological clusters. The overview of the entire clustering process, including testing cluster convergence with non-OSAS control patients and validation of *SAS*_Score_, is presented in Fig. 5.

For a network (*V*, *E*), a layout algorithm running in an Euclidean *k*-dimensional space *R*^*k*^ places each vertex *v* ∈ *V* to a corresponding position *p*_*v*_ ∈ *R*^*k*^ and assigns an Euclidean distance |*p*_*v*_ − *p*_*w*_| to each edge [*v*, *w*] ∈ *E*.

In particular, energy model layout algorithms are developed as attraction–repulsion (A-R) force systems (Noack, 2009). As such, in an A-R system, adjacent vertices attract while all the other pairs of vertices repulse; this is the emerging mechanism which leads to the formation of groups of vertices with dense connections that we interpret as communities or clusters. The A-R force values are proportional to the power (*A* or *R*) of the Euclidean distances between the nodes: the attraction between adjacent vertices *v* and *w* is ${{|}{p}_{v}-{p}_{w}{|}}^{A}\cdot \overrightarrow {{p}_{v},{p}_{w}}$, and the repulsion between any 2 vertices *v*, *w* ∈ *V* is ${{|}{p}_{v}-{p}_{w}{|}}^{R}\cdot \overrightarrow {{p}_{v},{p}_{w}}$ ($\overrightarrow {{p}_{v},{p}_{w}}$ is the unit vector from *v* to *w*). To generate topological communities that are consistent with connection densities, thus having the advantage of emphasizing distinct communities and clusters (Jacomy et al., 2014), attraction between two nodes has to decrease with the Euclidean distance between the nodes, while repulsion has to increase with the Euclidean distance, therefore we have *A* ≥ 0 and *R* ≤ 0; such representative force-based layouts are the Fruchterman and Reingold model (*A* = 2, *R* =  − 1) (Fruchterman & Reingold, 1991), and the LinLog model (*A* = 0, *R* =  − 1) (Noack, 2003). For all A-R energy models, the resulted edge positions are determined by a local energy minima situation (Noack, 2009), as described in Eq. (1):

(1) \begin{eqnarray*}\min \left\{ \sum _{[v,w]\in E,v\not = w} \left( \frac{{{|}{p}_{v}-{p}_{w}{|}}^{A+1}}{A+1} - \frac{{{|}{p}_{v}-{p}_{w}{|}}^{R+1}}{R+1} \right) \right\} .\end{eqnarray*}

In addition to the layout algorithm, we used modularity-based network clustering (Girvan & Newman, 2002), a method that was proven to be effective in network medicine (Diez, Agustí & Wheelock, 2014; Faner et al., 2014). Network clustering consists of assigning each vertex *v* ∈ *V* to one of the disjoint vertex subsets (or clusters) *C*_*i*_, such that ∪_*i*_*C*_*i*_ = *V*. In our APN clustering approach, modularity classes *C*_*i*_ are represented with distinct colors. Because the APN is an unweighted network, the modularity of any clustering is defined in Eq. (2), where |*E*_*C*_*i*__| is the number of edges in cluster *C*_*i*_, |*E*| is the total number of edges in the network, *d*_*C*_*i*__ is the total degree^1^ 1The degree of a vertex represents the number of its incident edges.for nodes in cluster *C*_*i*_, and *d* is the total degree for all nodes in the network:

(2) \begin{eqnarray*}\sum _{{C}_{i}} \left( \frac{{|}{E}_{{C}_{i}}{|}}{{|}E{|}} - \frac{ \frac{1}{2} {d}_{{C}_{i}}^{2}}{ \frac{1}{2} {d}_{C}^{2}} \right) .\end{eqnarray*}

Noack demonstrated that energy-model layout algorithms produce topological clusters that are equivalent with those rendered by modularity-based clustering (Noack, 2009). However, force-directed layouts provide additional topological information about clusters. As such, for a more accurate analysis, it is recommended that both modularity clustering and force directed layouts are used (Noack, 2009; Jacomy et al., 2014; Udrescu et al., 2016).

## Results

### APN analysis

**Figure 6:**
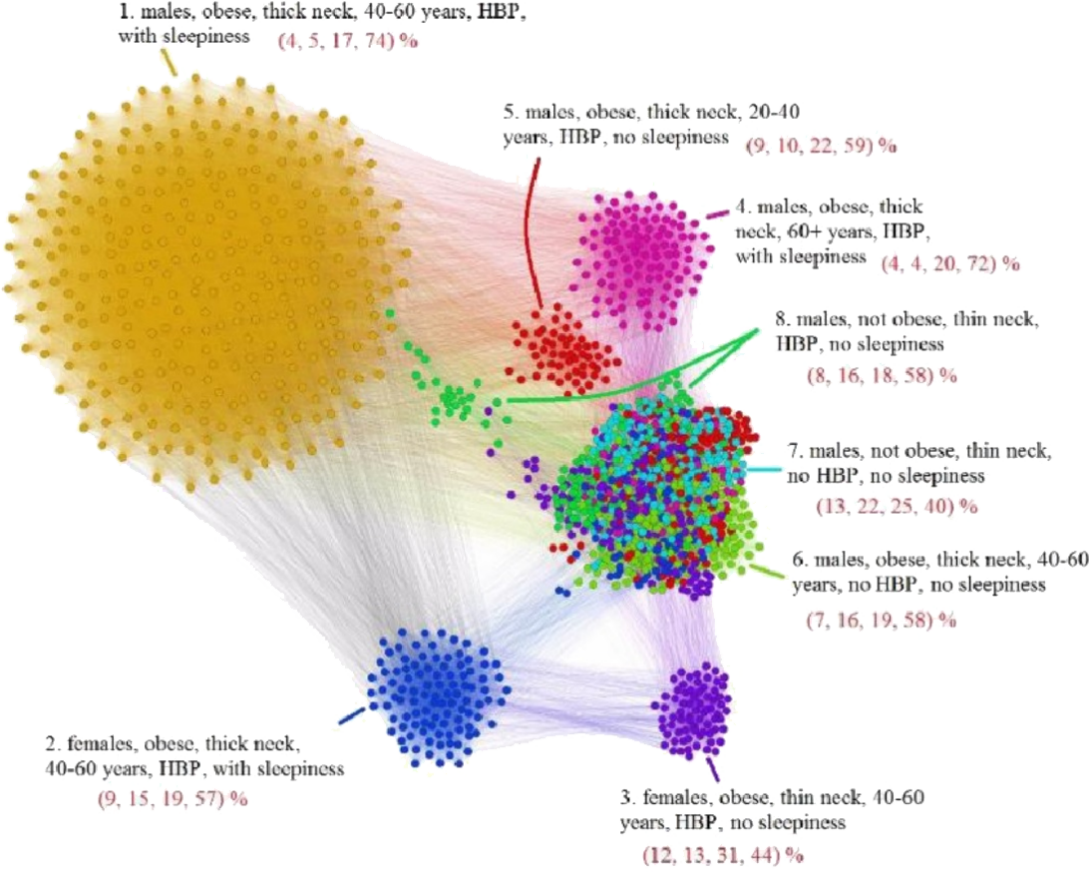
Apnea Patients Network (APN) obtained with data from the Apnea Patients Database (APD), according to the risk factor compatibility relationship, using our dual network clustering methodology (i.e., modularity classes and energy-model layouts). The assigned colors correspond to modularity classes, and the 8 topological clusters are indicated. For each topological cluster, statistics are provided in red (as percentages) for all AHI risk groups: low, mild, moderate, and severe, using the format (*L*, *Mi*, *Mo*, *Se*)% (e.g., in Cluster 2 the patients are distributed on risk groups as follows: 9% *L*, 15% *Mi*, 19% *Mo*, 57% *Se*). The risk group classification is made with *AHI* values that are obtained by actually performing polysomnography (PSG) and polygraphy.

The APN representation resulted from our clustering methodology is presented in Fig. 6, where the distinct colors correspond to distinct modularity classes, and the well-defined topological clusters are explained accordingly. In Fig. 6, we interpret the eight topological clusters as distinct phenotypes, and provide the risk factors prevalence as percentages (*L*, *Mi*, *Mo*, *Se*)% for each such cluster/phenotype.

### Non-OSAS Patients Network (NPN) analysis

**Figure 7:**
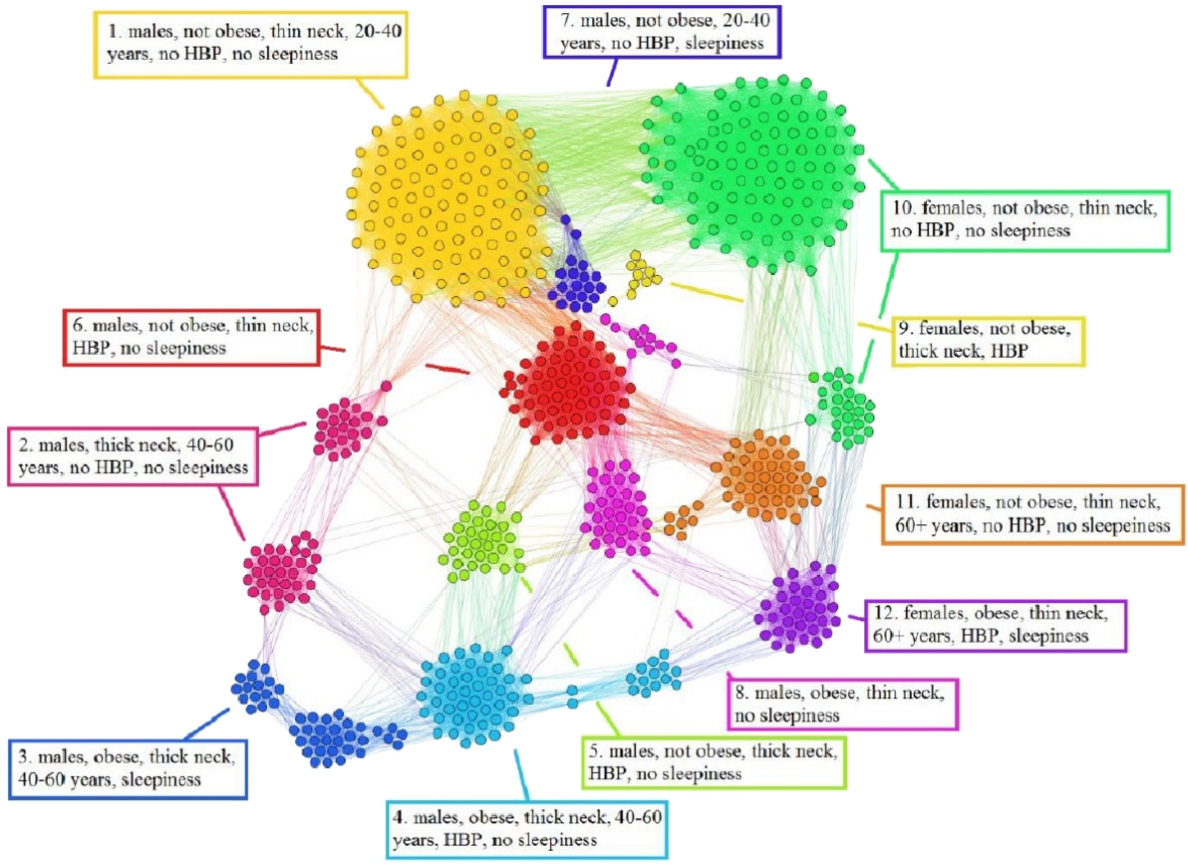
Non-OSAS Patients Network (NPN), obtained with data from the non-OSAS database (NAD) control population database. The colors correspond to 12 modularity classes. Also, the energy-model layout Force Atlas 2 generates 12 topological clusters, which are described in terms of risk factors and their correspondence with the modularity classes.

Using the information from the 611 people non-OSAS database (NAD), we employ the same procedure as for the APN from Fig. 6. The NAD represents the control population, consisting of people that are not diagnosed with OSAS. The result of applying our methodology on NAD patients is presented in Fig. 7, where the colors correspond to distinct modularity classes; at the same time, topological communities rendered with the energy-model layout Force Atlas 2 are indicated and explained.

Upon visual inspection, Fig. 7 suggests that in the non-OSAS control population there are more patterns of risk factors association, which leads to a number of 12 topological clusters and modularity classes that are not correlated with OSAS or AHI risk groups. As such, according to our network-based methodology, it occurs that the six considered risk factors consistently converge only for the individuals with OSAS.

### Description of phenotypes

**Figure 8:**
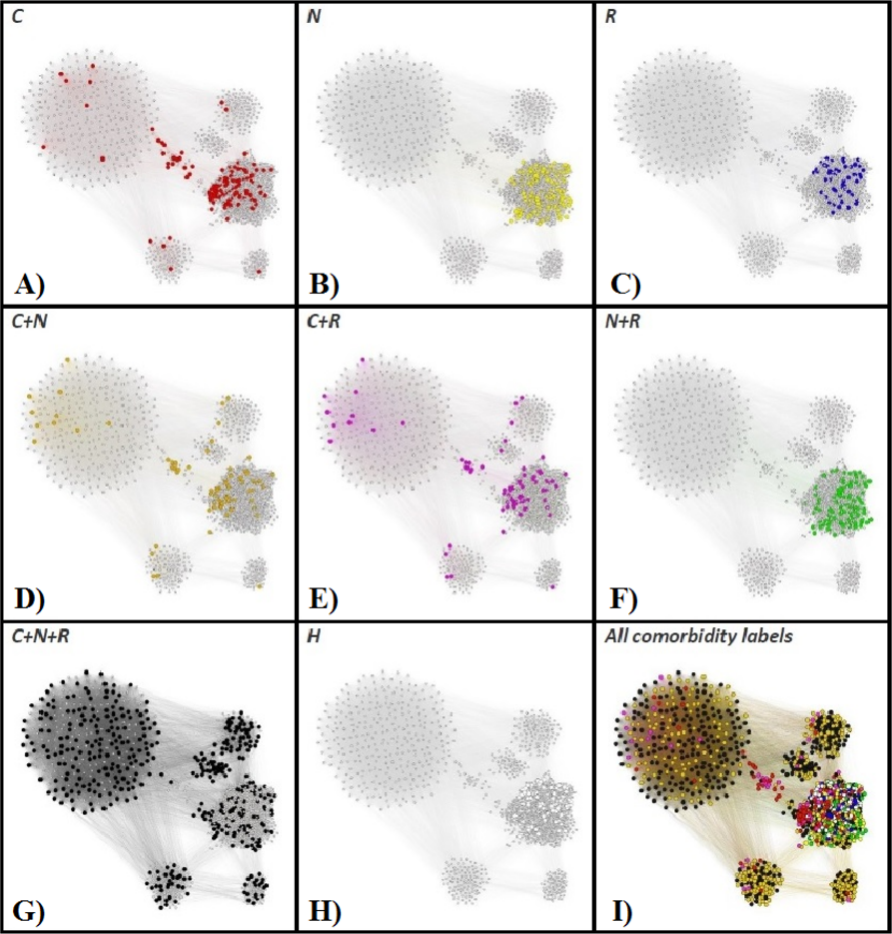
The Apnea Patients Network (APN), with highlighted individual comorbidities and associations of comorbidities. The red nodes correspond to patients that have only cardiovascular comorbidities (*C* in A), the yellow nodes to only nutrition-related (*N* in B), and blue nodes represent OSAS patients with only respiratory comorbidities (*R* in C). Patients with overlapping comorbidity types are represented within the APN as follows: orange nodes correspond to cardiovascular plus nutritional comorbidities (*C* + *N* in D), purple nodes to cardiovascular plus respiratory comorbidities (*C* + *R* in E), green nodes to nutritional plus respiratory comorbidities (*N* + *R* in F), and black nodes to the superposition of cardiovascular, nutritional and respiratory comorbidities (*C* + *N* + *R*, G). The OSAS patients without known comorbidities are highlighted in the APN as white nodes (*H* in H). We also provide the APN where all nodes are labeled according to their comorbidity or comorbidities overlapping in I.

In order to have a clear characterization of our rendered phenotypes, we are tracking the OSAS comorbidities (as recorded in the APD) within the APN. To this end, we consider the comorbidity types: *cardiovascular* (e.g., hypertension or stroke), *nutritional* (e.g., obesity or diabetes), and *respiratory*-related (e.g., COPD or asthma). Figure 8 presents the highlighted comorbidities within the APN by using distinct colors for comorbidity types that appear individually, as well as for comorbidity type overlaps (cardiovascular + nutritional, cardiovascular + respiratory, nutritional + respiratory, cardiovascular + nutritional + respiratory), and patients without known comorbidities.

In light of comorbidity and AHI risk groups statistics provided in Table 2 for each cluster resulted from our network analysis (as illustrated in Figs. 6 and 8), we characterize the phenotypes as follows:

• **Phenotype 1:** Mostly patients within the *Se* AHI risk group, which are generally obese males with thick neck, high blood pressure, sleepiness, and age between 40 and 60 years. For a large majority of these patients, all comorbidity types overlap.
• **Phenotype 2:** The large majority of these patients have *Mo* and *Se* apnea forms; they are obese females with thick neck, high blood pressure, sleepiness, age between 40 and 60 years. In this phenotype there are no patients with only respiratory comorbidities and only few of them have single nutritional comorbidities.
• **Phenotype 3:** The patients have mostly *Mo* and *Se* apnea, but there are less *Se* forms in comparison with other phenotypes; they are obese females with thin neck, high blood pressure, no sleepiness, and age between 40 and 60 years. This phenotype does not contain patients with only respiratory comorbidities.
• **Phenotype 4:** Mostly *Se* patients; however, there is a significant number of *Mo* individuals, which are generally obese males with thick neck, high blood pressure, sleepiness, and over 60 years. In this phenotype only a few patients have the single respiratory comorbidities type.
• **Phenotype 5:** Mostly *Se*, *Mo*, and *Mi* patients, which are obese young males with thick neck, high blood pressure, no sleepiness, and age between 20 and 40. In this phenotype, almost all patients have nutritional comorbidities or comorbidity overlaps that include the nutritional type.
• **Phenotype 6:** Consists of mostly *Mo* and *Se* apnea; the patients with this phenotype are generally obese males with thick neck, no high blood pressure, no sleepiness, and middle aged (40–60 years old). Their comorbidities are mostly nutritional-related (either single nutritional comorbidity or an association of comorbidities that contains the nutritional type).
• **Phenotype 7:** Patients with mostly *Mo* and *Se* apnea, but with less *Se* forms in comparison with other phenotypes; this phenotype’s patients are generally males of all ages with thin neck, no high blood pressure, and no sleepiness. The majority of these patients have no comorbidities; however, those who have a comorbidity tend to have respiratory-related problems.
• **Phenotype 8:** Patients mostly within *Se*, *Mo*, and *Mi* AHI risk groups; they are males from all age groups with thin neck, no sleepiness, but with high blood pressure. These patients tend to have a single cardiovascular comorbidity type or an association of comorbidities that include the cardiovascular type.

### OSAS risk prediction with *SAS*_Score_

Classifying any new patient in one of the phenotypes can be performed by adding the new patient to the APN and then running the modularity class and force-directed layout algorithms in Gephi one more time. However, during the screening process, physicians are frequently unable to perform these rather complex and time consuming steps (i.e., manipulation of databases and managing Gephi plugins), because of the obvious constraints.

**Table 2:** Description of the eight relevant Apnea Patients Network (APN) phenotypes. The phenotypes (Ph) are listed with a short description in terms of most or least predominant AHI risk groups (low risk- *L*, mild- *Mi*, moderate- *Mo*, and severe- *Se*), significant combinations of the 6 objective parameters, and most/least predominant comorbidity types (cardiovascular- *C*, nutritional- *N*, respiratory- *R*, and without comorbidities- *H*) or comorbidity types overlaps (*C* + *N*, cardiovascular + nutritional; *C* + *R*, cardiovascular + respiratory, *N* + *R*, nutritional + respiratory; *C* + *N* + *R*, cardiovascular + nutritional + respiratory). For each phenotype, we provide the corresponding percentages for comorbidity types and comorbidity type associations, as well as the percentage of patients pertaining to one of the AHI risk groups; the boldface entries correspond to representative values, in terms of simple majority. In phenotype descriptions, HBP stands for high blood pressure.

Ph.	Description	Comorbidity types and associations (%)								AHI risk groups (%)			
		*C*	*N*	*R*	*C* + *N*	*C* + *R*	*N* + *R*	*C* + *N* + *R*	*H*	*L*	*Mi*	*Mo*	*Se*
1	Mostly *Se* with *C* + *N* + *R* comorbidities	2.38	–	–	32.65	3.4	–	**61.57**	–	4	5	17	74
2	Mostly *Mo* and *Se*, thick neck females, no *R*, few *N*	6.49	0.65	–	**61.04**	3.9	0.65	27.27	–	9	15	19	57
3	Mostly *Mo*, *Se*, thin neck females, no *R*	13.66	10.24	–	**48.29**	1.95	2.93	22.93	–	12	13	31	44
4	Mostly *Mo* and *Se*, elderly males with thick neck, few *R*	6.52	3.62	–	**36.96**	5.8	3.62	**44.48**	–	4	4	20	72
5	Mostly *Mi*, *Mo*, *Se*, obese young males, mostly *N*	2.47	**18.52**	1.23	19.75	3.09	**20.99**	32.72	1.23	9	10	22	59
6	Mostly *Mo*, *Se*, 40–60 yrs. obese males, no HBP, mostly *N*	2.58	**33.55**	5.81	10.97	1.29	**31.61**	7.74	6.45	7	16	19	58
7	Less *Se*, non-obese, thin neck, no HBP males, mostly *H*, some *R*	8.84	0.68	27.21	0.68	4.76	–	–	**57.83**	13	22	25	40
8	Less *Se*, non-obese, thin neck, HBP males, mostly *C*	**38.79**	2.59	–	9.48	**28.45**	2.59	18.1	–	8	16	18	58

In order to deal with this problem, we propose a simplified solution for classifying *de novo* patients using a computer algorithm that is implemented as a web-based application. As such, we employ supervised machine learning in order to classify any new person in one of the eight validated phenotypes, based on the six relevant parameters. To this end, we choose decision tree learning, because decision trees are easy to use, quick, and intuitive for medical personnel. We use R and available recursive tree partitioning libraries from R Studio in order to perform recursive tree partitioning, thus generating a phenotype classification tree (Therneau et al., 2010; Hothorn, Hornik & Zeileis, 2006).

Given the eight phenotypes determined with our network procedure, and the features extracted from them, we label each patient from the APD with the cluster/phenotype to which it pertains. From this point, we employ supervised learning methods and then test them in order to chose the tree which provides the highest reliability while maintaining a reasonable complexity.

**Figure 9:**
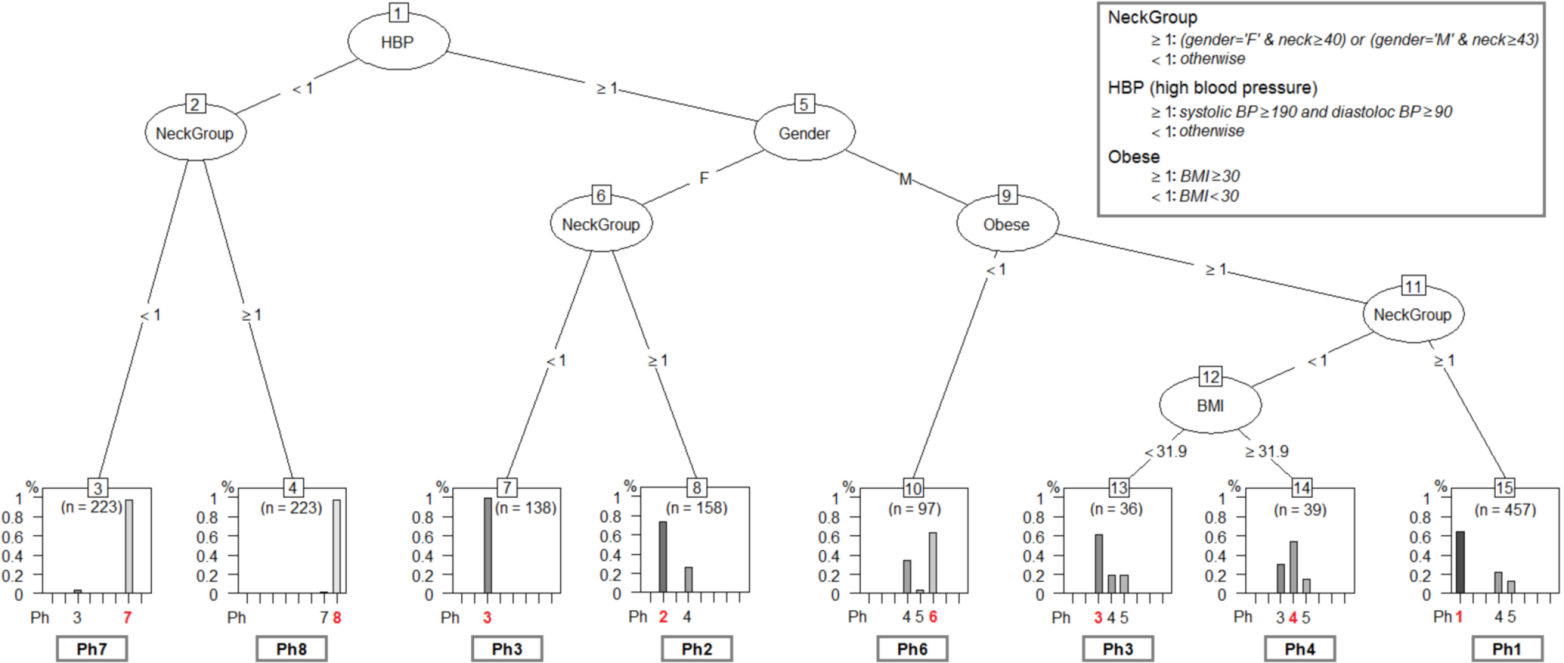
The phenotype classifying tree, obtained by running *evtree* on patients from APD. Each new person is assigned to one of the eight phenotypes, denoted as *Ph*1–*Ph*8 (dominant phenotypes are emphasized). Decisions are made according to some of the six relevant parameters (gender-*G*, age- *A*, body mass index-*BMI*, systolic blood pressure-*SBP*, diastolic blood pressure-*DBP*, Epworth sleepiness score-*ESS*), but also according to variables computed from the six parameters (High Blood Pressure-*HBP*, Neck Group-*NG*, Obesity-*Ob*), as described in the figure legend.

The tested mining algorithms are: recursive partition tree, conditional inference tree, evolutionary tree, oblique tree, maptree, naive Bayes, random forest and linear regression (Therneau et al., 2010; Hothorn, Hornik & Zeileis, 2006; Grubinger, Zeileis & Pfeiffer, 2011). The algorithm test procedure relies on applying the resulted decision trees on *de novo* patients from the Test Database (TD). We take all patients in TD and assign each of them to a phenotype with the classification tree. In parallel, we add the TD patients to the APD, and rerun the entire layout clustering procedure. Indeed, we obtain the same eight patient phenotypes, and we consider the phenotype labels that are assigned to patients in TD by our network clustering procedure as being the reference (i.e., correct phenotype assignation). Therefore, we quantify the efficiency of each decision tree by comparing with the reference represented by the network-based classification. The test results indicate the evolutionary tree (evtree) Grubinger, Zeileis & Pfeiffer (2011), which is given in Fig. 9, as the best method for our classification problem.

The classification tree in Fig. 9 uses the six relevant parameters (gender- *G*, age- *A*, body mass index- *BMI*, systolic blood pressure- *SBP*, diastolic blood pressure- *DBP*, Epworth sleepiness score- *ESS*), but also variables that are computed from the six parameters (High Blood Pressure- *HBP*,Neck Group- *NG*, Obesity- *Ob*) according to Eqs. (3)– (5):

(3) \begin{eqnarray*}& & HBP= \left\{ \begin{array}{@{}lll@{}} \displaystyle 1&\displaystyle \text{if}&\displaystyle SBP\geq 140 \text{and} DBP\geq 90\\ \displaystyle 0&\displaystyle \text{otherwise}&\displaystyle \\ \displaystyle \end{array} \right. \end{eqnarray*}

(4) \begin{eqnarray*}& & NG= \left\{ \begin{array}{@{}lll@{}} \displaystyle 1&\displaystyle \text{if}&\displaystyle \left( G=\text{female and} NC\geq 40 \right) \text{or} \left( G=\text{male and} NC\geq 43 \right) \\ \displaystyle 0&\displaystyle \text{otherwise}&\displaystyle \\ \displaystyle \end{array} \right. \end{eqnarray*}

(5) \begin{eqnarray*}& & Ob= \left\{ \begin{array}{@{}lll@{}} \displaystyle 1&\displaystyle \text{if}&\displaystyle BMI\geq 30\\ \displaystyle 0&\displaystyle otherwise&\displaystyle \\ \displaystyle \end{array} \right. \end{eqnarray*}

When we test the classification tree in Fig. 9 on patients from TD and compare the results against the reference classification (i.e., network-based), the resulted classification accuracy is 69.30%; the detailed prediction accuracy results for TD are given in Table 3.

**Table 3:** Prediction table for each phenotype and associated classification error, as obtained by employing evtree on TD. Each line in the table shows (in bold characters) how many patients in TD are correctly classified in the corresponding phenotype.

		Patients actually belonging to phenotype								
		Ph 1	Ph 2	Ph 3	Ph 4	Ph 5	Ph 6	Ph 7	Ph 8	Error
Patients predicted in	Ph 1	**294**	0	0	103	60	0	0	0	35.67%
	Ph 2	0	**116**	0	42	0	0	0	0	26.58%
	Ph 3	0	0	**163**	11	12	1	0	0	12.36%
	Ph 4	0	0	8	**17**	1	0	0	0	34.61%
	Ph 5	0	0	0	0	**0**	0	0	0	100%
	Ph 6	0	0	0	33	3	**61**	0	0	37.11%
	Ph 7	0	0	7	0	0	0	**216**	0	3.14%
	Ph 8	0	0	0	2	0	0	3	**218**	2.24%

In order to generate our *SAS*_Score_ from the classification tree in Fig. 9, we follow the next sequence of steps:

1. Perform anthropometric measurements on each new patient.
2. Classify each patient in one of the eight phenotypes (using the classification from Fig. 9).
3. Refer to cluster normalization in order to get phenotype parameter averages from Table 4.
4. Compute *SAS*_Score_ using Eq. (6).

Therefore, having the six recorded parameters for any new patient that has to be evaluated, and the average values $BM{I}_{a}^{\mathrm{Cluster}}$, $N{C}_{a}^{\mathrm{Cluster}}$, $SB{P}_{a}^{\mathrm{Cluster}}$, $DB{P}_{a}^{\mathrm{Cluster}}$, $ES{S}_{a}^{\mathrm{Cluster}}$ with $Cluster\in \left\{ 1,2,\ldots ,8 \right\} $ as presented in Table 4, the *SAS*_Score_ is automatically computed by our computer application according to Eq. (6), then correspondingly displayed by the web-based interface.

(6) \begin{eqnarray*}SA{S}_{\mathrm{Score}}= \frac{BMI}{BM{I}_{a}^{\mathrm{Cluster}}} + \frac{NC}{N{C}_{a}^{\mathrm{Cluster}}} + \frac{1}{2} \left( \frac{SBP}{SB{P}_{a}^{\mathrm{Cluster}}} + \frac{DBP}{DB{P}_{a}^{\mathrm{Cluster}}} \right) + \frac{ESS}{ES{S}_{a}^{\mathrm{Cluster}}} .\end{eqnarray*}

**Table 4:** Average values for the relevant parameters (body mass index- *BMI*, neck circumference- *NC*, systolic blood pressure- *SBP*, diastolic blood pressure- *DBP*, Epworth sleepiness score- *ESS*), which are computed for each of the eight clusters in Fig. 6.

	Cluster/Phenotype							
	1	2	3	4	5	6	7	8
$BM{I}_{a}^{\mathrm{Cluster}}$	36.83	37.54	31.88	33.75	34.99	32.93	22.74	28.67
$N{C}_{a}^{\mathrm{Cluster}}$	47.66	43.9	35.91	46.79	43.17	43.66	33.39	37.36
$SB{P}_{a}^{\mathrm{Cluster}}$	143.96	145.32	139.48	140.56	133.74	125.46	119.66	134.55
$DB{P}_{a}^{\mathrm{Cluster}}$	89.46	87.61	85.80	84.28	84.43	80.75	73.16	84.41
$ES{S}_{a}^{\mathrm{Cluster}}$	11.49	10.77	7.74	10.10	8.58	9.50	6.28	8.90

*SAS*_Score_ values are ≥1 and generally <7; this range of values can further serve to classify patients as being at risk of developing OSAS or not. A patient with *SAS*_Score_ > *threshold* is considered at risk, whereas *SAS*_Score_ ≤ *threshold* means that there is no OSAS risk. In order to attain the main objective of our paper, namely to make population-wide OSAS monitoring and screening effective, we consider the higher specificity as being more important, so we choose *threshold* = 3.9, which determines a sensitivity of 0.8025 and a specificity of 0.4189. Taken together, these results obtained with the TD, consisting of *de novo* patients only, suggest that our *SAS*_Score_ significantly outperforms STOP-BANG in terms of specificity (i.e., it is 2.34 times better), while remaining only slightly worse than STOP-BANG in terms of sensitivity (8.2% decrease). We consider these results as being particularly relevant, because the distribution of *AHI* in the TD is notably different from the distribution of *AHI* in APD (see Fig. 3 panels A and B).

### Validation of clustering consistency

In this subsection, we verify that rendering the clusters in Fig. 6 is not mere serendipity, and it is not induced by some fortunate heterogeneity of patients. To this end, we perform random shuffling and bootstrapping test investigations.

Because our clustering methodology starts with a random state, namely it starts with the raw network where nodes are randomly placed and the links have corresponding lengths (see Fig. 5), our first shuffling test consists of running the procedure in Fig. 5 many times in order to see if we get statistically consistent results. Therefore, we run our dual clustering procedure 100 times on the same APD, and then measure the distribution of anthropometric values for each phenotype. The result of our random shuffling is given in Table 5 which presents average anthropometric measurements with standard deviations (expressed as percentages) for the each APN cluster/phenotype after 100 runs. Indeed, the deviations from the average values are very small, emphasizing the consistency of our clustering procedure in Fig. 5.

**Table 5:** Average anthropometric measurements with standard deviations (expressed as percentages $ \left( \text{%} \right) $) for the anthropometric parameters (Age *A*, *BMI*, Neck Circumference *NC*, High Blood Pressure *HBP*, Epworth Sleepiness Score *ESS*, Apnea-Hypopnea Index *AHI*) in the APN clusters/phenotypes after 100 runs (for instance, the average age in cluster 2 is 54.11 years with a standard deviation of 3.51%).

Cluster	*A*	*BMI*	*NC*	*HBP*	*ESS*	*AHI*
1	51.89 ± 0	36.83 ± 0	47.66 ± 0	100% ± 0	11.49 ± 0	50.75 ± 0
2	54.11 ± 3.51	37.73 ± 0.86	44.13 ± 0.47	92% ± 5.22	10.59 ± 0.99	38.3 ± 0.49
3	56.14 ± 1.87	31.88 ± 1.27	35.87 ± 1.13	87% ± 5.77	7.8 ± 2.45	30.92 ± 3.09
4	66.47 ± 0.28	33.84 ± 1.55	44.15 ± 2.46	90% ± 6.78	10.02 ± 1.64	42.99 ± 3.3
5	34.54 ± 1.34	35.09 ± 1.53	44.52 ± 2.39	51% ± 5.27	8.94 ± 3.26	49.66 ± 4.94
6	51.23 ± 2.45	32.94 ± 1.66	45.23 ± 3.08	0% ± 0	9.85 ± 2.82	44 ± 4
7	43.59 ± 2.62	24.6 ± 5.77	33.01 ± 1.35	7% ± 10.57	6.46 ± 2.51	25.93 ± 2.9
8	55.92 ± 4.47	27 ± 3.55	39.96 ± 4.51	96% ± 10.42	8.75 ± 3.64	34.29 ± 2.66

Our second test approach entails generating test APDs from the original patient dataset, in order to perform bootstrapping. To do so, we generate 10 new APD datasets with the same number of patients as the original APD by randomly selecting patients from the original APD database. Therefore, in the test APDs, some of the original patients may be missing, while others may be present two or more times. Next, we apply the same clustering methodology from Fig. 5 and find that the same phenotypes emerge.

The characteristics of the original APN phenotypes from Fig. 6 are provided in Table 6; Table 7 shows the averaged characteristics, for each cluster, over the randomized 10 APNs obtained by bootstrapping. In Table 7 distinct character types suggest how close (bold and normal characters) or how far (grey italics) are the phenotype characteristics resulted from bootstrapping from the original APN values.

**Table 6:** Defining phenotype characteristics given as majority values with corresponding percentages, as measured in the original APN communities.

Cluster	*G*	*Ob*	*TN*	*AG*	*HBP*	*SLP*
1	M (100%)	1 (100%)	1 (100%)	3 (100%)	1 (100%)	1 (61%)
2	F (100%)	1 (96%)	1 (100%)	3 (92%)	1 (100%)	1 (56%)
3	F (80%)	1 (81%)	0 (100%)	3 (69%)	1 (96%)	0 (70%)
4	M (100%)	1 (82%)	1 (77%)	4 (100%)	1 (99%)	1 (52%)
5	M (100%)	1 (96%)	1 (83%)	2 (100%)	1 (100%)	0 (64%)
6	M (88%)	1 (87%)	1 (100%)	3 (57%)	0 (100%)	0 (57%)
7	M (67%)	0 (61%)	0 (99%)	3 (50%)	0 (100%)	0 (77%)
8	M (98%)	0 (100%)	0 (55%)	3 (84%)	1 (100%)	0 (58%)

**Table 7:** Characteristic features of phenotypes averaged over 10 randomized APNs. Majority average percentages for the dominant values are rounded to the nearest integer, while the colours represent how close these averages are to the measurements on the original APN from Table 6: bold entries correspond to very close matches (percentage difference ≤ 6%), normal character entries correspond to a good match (6% < percentage difference ≤15%), while grey-italics table entries correspond to significant differences (>15%).

Cluster	*G*	*Ob*	*TN*	*AG*	*HBP*	*SLP*
1	**M (100%)**	**1 (100%)**	**1 (100%)**	**3 (100%)**	**1 (100%)**	**1 (60%)**
2	**F (100%)**	**1 (96%)**	**1 (100%)**	* 3 (74%)*	**1 (100%)**	**1 (60%)**
3	F (90%)	**1 (81%)**	**0 (100%)**	**3 (63%)**	1 (87%)	**0 (69%)**
4	**M (95%)**	**1 (86%)**	1 (88%)	**4 (100%)**	**1 (97%)**	**1 (53%)**
5	**M (97%)**	**1 (97%)**	**1 (86%)**	**2 (100%)**	1 (79%)	**0 (62%)**
6	M (95%)	**1 (87%)**	**1 (96%)**	* 3 (73%)*	**0 (100%)**	**0 (58%)**
7	* M (81%)*	**0 (64%)**	0 (89%)	* 3 (75%)*	**0 (99%)**	0 (70%)
8	* M (82%)*	0 (84%)	* 0 (80%)*	3 (68%)	**1 (100%)**	0 (71%)

The values from Table 7 indicate that the dominant characteristics of each cluster in the test APNs are very similar to the cluster characteristics from the original APN. As a result, even if we create new APDs with corresponding APNs by shuffling the patients from our dataset, the bootstrapping procedure yields the same phenotypes. The bootstrapping procedure reveals that some phenotypes emerge as more stable when randomized (e.g., clusters 1, 3, 4, 5), with a small offset compared to the original APN. The lack of convergence over multiple randomizations could be interpreted as a lack of representativeness as an OSAS phenotype. As such, clusters 2 and 6 are slightly less representative, and clusters 7 and 8 are notably variable, suggesting that the last two phenotypes may not be so well characterized, due to the reduced amount of patient data.

**Figure 10:**
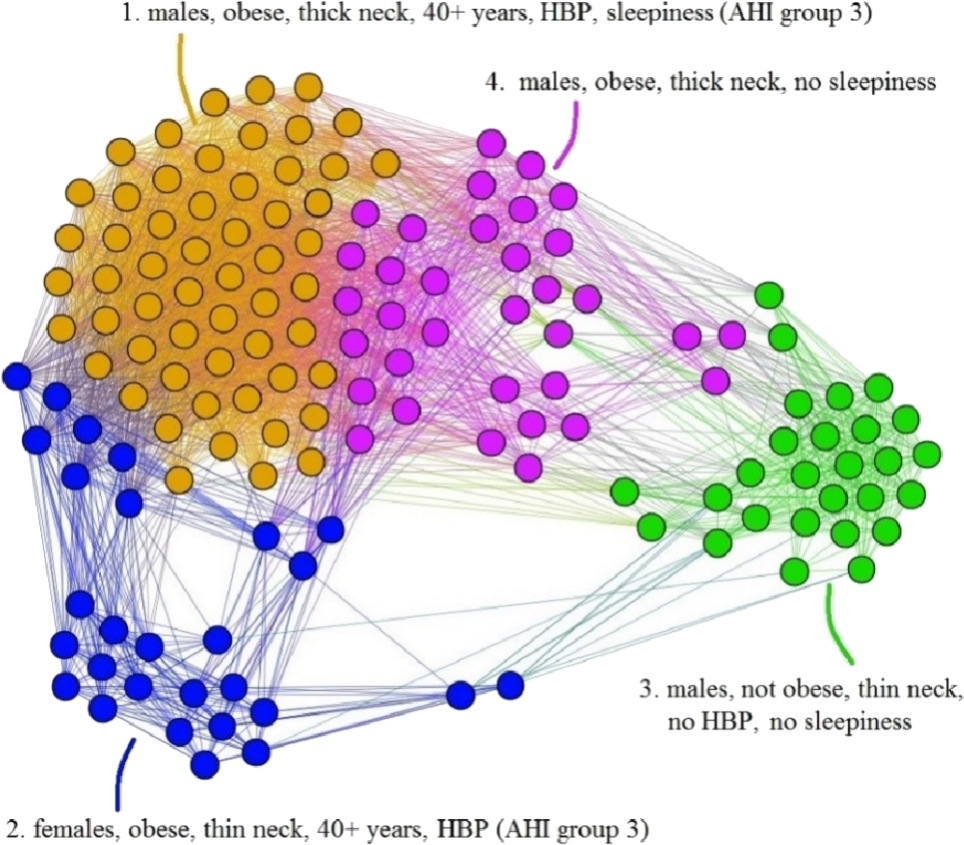
Representation of Test Database-TD patients, according to the risk factor compatibility, using the proposed network-based methodology. Although the number of patients is significantly smaller in comparison with the primary Apnea Patients Network-APN, the visual inspection reveals that the emerged clusters are similar to clusters from Fig. 6 (APN).

We also create a test patient network (TPN), similar to building the APN, and then apply our dual clustering methodology. The result is presented in Fig. 10; upon visual inspection it can be noticed that the clusters emerged in TPN are similar to the clusters from Fig. 6’s APN, even if the number of patients is significantly smaller in TD; this result suggests that the association and convergence of risk factors in OSAS patients is indeed a non-random, consistent process.

## Discussion

The proposed method is not the first to cluster apnea patients (Joosten et al., 2012; Vavougios et al., 2016; Ye et al., 2014), but to the best of our knowledge it is the first network-based approach used for clustering apnea patients. Another important feature is that our network-based methodology employs only easy-to-measure, objective clustering parameters (*AHI* is used only for phenotype evaluation). This way, our clustering methodology emphasizes the high complexity of OSAS phenotypes, from typical (cluster 1—yellow) to the less obvious ones (clusters 6–8).

When defining the eight apnea phenotypes, besides the force-directed layout, we also use modularity class clustering. In Fig. 6, the phenotypes based on modularity classes are generally consistent with the topological clusters resulted from applying the Force Atlas 2 layout. However, the visual inspection of Fig. 6 reveals that phenotypes 5, 6, 7, and 8 tend to spatially overlap; this tendency is much stronger for phenotypes 6, 7, and 8. Such a tendency for overlapping phenotypes that characterize patients with generally mild and moderate OSAS is also suggested by Joosten et al. (2012). This observation may indicate that these phenotypes are interrelated and generally hard to distinguish even in clinical practice. Still, some of the nodes in these clusters (e.g., cluster 8) have a clear tendency towards separation from the overlapping; this indicates that we probably need more patients/nodes, in order to completely segregate Cluster 8. As our APN will grow over time, the less convergent clusters 7 and 8 might become more representative. Indeed, the fact that even in the original APN from Fig. 6 clusters 7 and 8 present significant overlapping and that their topological segregation from other clusters is somehow fuzzy confirms the conclusion of our bootstrapping investigation. Nonetheless, we preferred to use the distinct modularity classes in conjunction with the topological clusters because they bring more information, i.e., more detail which can be useful for medical analysis.

From a medical standpoint, we note that our dual clustering method renders distinct male and female clusters; this observation is consistent with the state of the art medical literature which holds gender as a very important predictor of OSAS. For instance, in a 2009–2013 study on 272,705 patients from North America, referred for home sleep apnea testing, clinical OSAS features are found to be more common in males than females (Cairns, Poulos & Bogan, 2016). Other studies performed on 23,806 (Gabbay & Lavie, 2012), and 1,010 (Vagiakis et al., 2006) patients respectively, show clear differentiation between the two genders in terms of AHI distribution and severity.

In current practice, the commonly used score for predicting sleep apnea is STOP-BANG. In comparison with STOP-BANG, our *SAS*_Score_ significantly improves the prediction specificity (2.34 times better than STOP-BANG), while sensitivity is only slightly degraded. STOP-BANG has high sensitivity because it is a simple heuristic that was especially designed for perioperative patients, where it is essential to identify all potential risks associated with anaesthesia (including OSAS). To further emphasize the higher specificity of *SAS*_Score_, we mention that by using our score, only 34% people from NAD are found as at risk of developing OSAS. Moreover, as opposed to STOP-BANG (a fixed questionnaire that cannot be adjusted to specific patients characteristics), *SAS*_Score_ represents an adaptive methodology. Therefore, as the database grows, better sensitivity and specificity are expected. The classifying tree which leads to rendering *SAS*_Score_, as described in section *OSAS risk prediction with SAS*_Score_** represents a simplified application of our patient clustering/phenotyping method, but this method has the advantage of being applicable in offline conditions, which makes it amenable to clinical practice and population screening. All these considerations indicate *SAS*_Score_ as an appropriate tool for OSAS screening in large general populations.

Our network-based method represents an application on patients from a given geographical area, therefore we consider that it should be tested for other targeted populations. As such, the network analysis will render new, specific cluster average values (such as $BM{I}_{a}^{\mathrm{Cluster}}$, $N{C}_{a}^{\mathrm{Cluster}}$, $SB{P}_{a}^{\mathrm{Cluster}}$, $DB{P}_{a}^{\mathrm{Cluster}}$, $ES{S}_{a}^{\mathrm{Cluster}}$). Subsequently, *SAS*_Score_ values that are specific to the targeted population can be rendered with the *SAS*_Score_ equation. However, we do not expect that the phenotypes or *SAS*_Score_ will be significantly different for other populations, since the available medical studies, performed over diverse geographical areas (including a wide array of anthropometric characteristics), show that specific population traits are not particularly relevant for OSAS (Ralls & Grigg-Damberger, 2012; Lee et al., 2008; Villaneuva et al., 2005).

Eventually, due to its higher specificity, the *SAS*_Score_ can be integrated into a large area apnea screening and monitoring procedure, which aims at specifically discovering typical severe cases (easy to investigate with portable devices), without overcrowding sleep laboratories with false positive cases. This way, efficient personalized patient processing can be achieved by making use of prioritization according to the predicted severity level. For instance, this method can be a useful tool for sleep apnea screening in large population categories, such as professional drivers since, at the European level, the new 2014/85/EU directive regarding professional drivers is recommended from January 2016.^2^ 2Commission Directive COMMISSION DIRECTIVE 2014/85/EU of 1 July 2014 amending Directive 2006/126/EC—European Parliament and the Council on driving licences.In this context, our website http://sasscore.appspot.com is a good example of a large-area, accessible OSAS risk prediction tool. Indeed, *SAS*_Score_ can be conveniently computed in both clinical and population-monitoring practices, due to the fact that it is implemented as easy-to-use smartphone and web-based applications (https://play.google.com/store/apps/details?id=aerscore.topindustries.aerscore&hl=en and www.pneumoresearch.ro). To this end, processing data and obtaining the prediction score requires less than 1 min per individual.

## Conclusion

This paper proposes a new OSAS patients clustering method based on complex network analysis, which leads to identifying OSAS phenotypes. This innovative network medicine approach is extended in order to compute *SAS*_Score_, a predictive score for OSAS based on 6 easy-to-measure, objective parameters. The proposed method uses big data, complex networks analysis in order to achieve better specificity in OSAS prediction. As such, our *SAS*_Score_ can conveniently be used in conjunction with the existing questionnaires for better OSAS prevention through population screening and monitoring, thus paving the way for a personalized patient management process.

##  Supplemental Information

10.7717/peerj.3289/supp-1 Supplemental Information 1 Patient databases used in the paper This archive contains 2 xlsx files with the following databases: Apnea Patients Database (APD), Test Database (TD), and Non-osAs Database (NAD).

10.7717/peerj.3289/supp-2 Supplemental Information 2 Gephi files with the networks in the paper Archive containing the files with layout representations for the networks that are described in the paper. In order to visualize the .gephi files, the Gephi package has to be downloaded from https://gephi.org/ and installed.

## Additional Information and Declarations

### Data Availability

The following information was supplied regarding data availability: The raw data has been supplied as  Supplementary Files.
